# Diagnostic and therapeutic potential of RNASET2 in Crohn’s disease: Disease-risk polymorphism modulates allelic-imbalance in expression and circulating protein levels and recombinant-RNASET2 attenuates pro-inflammatory cytokine secretion

**DOI:** 10.3389/fimmu.2022.999155

**Published:** 2022-11-16

**Authors:** Eva Biener-Ramanujan, Florian Rosier, Simon G. Coetzee, Dermot D. P. McGovern, Dennis Hazelett, Stephan R. Targan, Rivkah Gonsky

**Affiliations:** ^1^ Inflammatory Bowel & Immunobiology Research Institute, Cedars-Sinai, Los Angeles, CA, United States; ^2^ Department of Biomedical Sciences, Cedars−Sinai Medical Center, Los Angeles, CA, United States

**Keywords:** Crohn’s disease, allelic imbalance, inflammation, human, gene regulation, cytokine

## Abstract

Ribonuclease T2 gene (RNASET2) variants are associated in genome wide association studies (GWAS) with risk for several autoimmune diseases, including Crohn’s disease (CD). In T cells, a functional and biological relationship exists between TNFSF15-mediated enhancement of IFN−γ production, mucosal inflammation and RNASET2. Disease risk variants are associated with decreased mRNA expression and clinical characteristics of severe CD; however, functional classifications of variants and underlying molecular mechanisms contributing to pathogenesis remain largely unknown. In this study we demonstrate that allelic imbalance of RNASET2 disease risk variant rs2149092 is associated with transcriptional and post-transcriptional mechanisms regulating transcription factor binding, promoter-transactivation and allele-specific expression. RNASET2 mRNA expression decreases in response to multiple modes of T cell activation and recovers following elimination of activator. In CD patients with severe disease necessitating surgical intervention, preoperative circulating RNASET2 protein levels were decreased compared to non-IBD subjects and rebounded post-operatively following removal of the inflamed region, with levels associated with allelic carriage. Furthermore, overexpression or treatment with recombinant RNASET2 significantly reduced IFN-γ secretion. These findings reveal that RNASET2 cis- and trans-acting variation contributed regulatory complexity and determined expression and provide a basis for linking genetic variation with CD pathobiology. These data may ultimately identify RNASET2 as an effective therapeutic target in a subset of CD patients with severe disease.

## Introduction

RNASET2 is the only human member of the Rh/T2/S family of acidic hydrolases. These endonucleases are highly conserved among the phyla from viruses to humans suggesting an important evolutionary function. Altered expression of RNASET2 is associated with cancer, autoimmune diseases, biological stress, apoptosis, and innate immune triggering (1–4). Additionally, RNASET2 contributes to cytoskeletal re-organization and caspase activation in response to oxidative stress (5, 6). The RNASET2 locus has been implicated by GWAS in susceptibility for vitiligo, Rheumatoid arthritis, Graves’ disease and Crohn’s disease (7–11). While RNASET2 is generally thought of as an extracellular ribonuclease, diverse biological roles have been ascribed for intracellular production and extracellular secretion of RNASET2. Mitochondrial localized RNASET2 facilitates ribosomal RNA decay and processing of telomerase RNA and lysosomal RNASET2 is a key component of the TLR8 RNA-sensing mechanism (12–15). Tumor cell secretion of RNASET2 in the extracellular milieu triggers cellular migration and activation of innate immune cells and extensive cytoskeletal-actin remodeling (16). In fact, several studies point to an oncosuppressive role for RNASET2 *via* monocyte/macrophage recruitment, activation and polarization (16–19). Somewhat surprisingly, the ribonuclease catalytic activity of RNASET2 may not be required for its oncosuppressive activity or stress mediated cell death ( (5, 20) The efficacy of human recombinant RNASET2 in inhibiting tumor growth, cytoskeletal reorganization and inflammatory response in several disease models supports the potential for RNASET2 as a soluble therapeutic drug (6, 18, 21, 22). While some of these studies have compared expression levels of RNASET2 mRNA transcript and protein in diseased tissue, studies of circulating RNASET2 protein levels are lacking.

Despite the association of RNASET2 with disease, its functional role in pathogenesis remains poorly defined. Moreover, the cis-and trans-regulatory regions of RNASET2 are largely uncharacterized. Both RNASET2 and TNFSF15 have been identified as IBD susceptibility loci (10, 11). TL1A, the protein encoded by TNFSF15, is a key mediator of mucosal inflammation (23, 24). In IBD patients, elevated TL1A levels correlate with TNFSF15 genotype and disease severity. Crohn’s disease (CD) patients with elevated serum/tissue levels of TL1A have increased risk of developing fibrostenosing/stricturing disease behavior (25, 26). We have previously shown in T cells that there is a functional and biological relationship between RNASET2 and TL1A-mediated enhancement of IFN−γ production, a key mediator of mucosal inflammation (27). We further demonstrated that down-modulation of RNASET2 expression occurs following TL1A mediated T-cell stimulation. RNASET2 disease-risk variants are functionally associated with a decrease in its expression in peripheral and mucosal tissues and with DNA hypermethylation. Furthermore, in CD, RNASET2 disease-risk variants are associated with a more complicated/resistant disease course. Thus, T cell mediated inflammation resulting in a decrease in RNASET2 expression might underlie complicated CD phenotype.

In the present study, we extend our previous findings to determine the molecular pathways regulating RNASET2 expression to better understand the associated disease pathobiology. We identify the contribution of cis- and trans-acting variants in regulating RNASET2 expression and demonstrate RNASET2 allelic imbalance in transcription factor complex formation, promoter transactivation and allele-specific expression. Decreased expression of RNASET2 is a general feature accompanying T-cell activation. We report for the first time to our knowledge, a decreased level of circulating RNASET2 protein associated with risk allelic carriage suggesting it may have application as a surrogate T-cell activation marker. In fact, circulating RNASET2 levels differed when comparing CD patients to healthy control subjects. Furthermore, in CD patients there was a significant increase in circulating RNASET2 when comparing pre-operative to post-operative levels after surgical resection of inflamed intestinal tissue. Finally, we demonstrated *in-vitro* the therapeutic feasibility of recombinant RNASET2 to attenuate proinflammatory cytokine secretion.

## Materials and methods

### Study subjects

Subjects were recruited through the MIRIAD IBD Biobank at the Cedars-Sinai F. Widjaja Foundation Inflammatory Bowel and Immunobiology Research Institute. Control subjects had no known personal or family history of autoimmune disease or IBD. Informed consent (approved by the Cedars-Sinai Institutional Review Board) was provided by all participating subjects. Genotyping for RNASET2 rs2149092 as previously described (27).

### Isolation of PBMC, blood plasma and CD4^+^ T cells

Peripheral blood mononuclear cells (PBMC) and plasma were isolated from healthy volunteers or IBD patients by separation on Ficoll-Hypaque gradients. CD4^+^ T cells were isolated using negative selection with magnetic beads (Stemcell Technologies, Vancouver, BC, Canada) and were at least 95% pure.

### Gel mobility electrophoretic shift assay

Nuclear extract proteins isolated from CD4^+^ T cells (3–6 µg) were incubated at 25°C with 0.25 mg/ml poly (dI-dC), in 20% glycerol, 5 mM MgCl2, 2.5 mM EDTA, 2.5 mM DTT, 250 mM NaCl, 50 mM Tris pH 7.5 for 10 min. Oligonucleotides 5′-IRD700-labeled (Integrated DNA Technology, Coraville, IA) (250 fmol) were then added and the binding reactions incubated for an additional 30 min. Specificity was determined by the addition of excess unlabeled oligonucleotide as competitor. Gel supershift assays were conducted by the addition of ETS1 Abs (Santa Cruz Biotechnology, Santa Cruz, CA) in the binding mixture prior to adding labeled oligonucleotide. The DNA–protein complexes were separated from unbound probe on a pre-run native 6% polyacrylamide gel in low ionic strength buffer (22.3 mM Tris pH 7.4, 22.3 mM Borate, 0.5 mM EDTA pH 8.0) and analyzed with Odyssey infrared imaging system (Li-Cor Biosciences). The oligonucleotides used were: rs2149092 C/T 5’CTTGTCACTTCC/TTCCTGTACTG3’.

### Quantitative PCR

Total RNA from PBMC or CD4^+^ T cells was extracted using RNAeasy plus mini kit (Qiagen Germantown MD). cDNA was synthesized from 1μg RNA using Omniscript kit (Qiagen) and used for qPCR analysis (FastStart SYBR Green reagent (Sigma St. Luis, MO) with the following primer sets: RNASET2-Fwd 5’-GAGTGATACCCAAAATCCAGT-3’; RNASET2-Rev 5’-GCTTAGTGAGGCACAGTTCT-3’; ETS1-Fwd 5’-ATGAATGGAGCAGCCCTCTG-3’; ETS1-Rev 5’-ACTCCGATGGTGGAACACAC-3’; ELF2-Fwd 5’-GTTGGCCGTAAACCAAAGACC-3’; ELF2-Rev 5’-AGACAGCCTTTGAATCCACCA-3’; ACTB Fwd: 5’-CGTGCTGCTGACCGAGG-3’, ACTB Rev: 5’-AAGGTCTCAAACATGATCTGGGT-3’.

### Allele specific expression

ASE was assayed according to quantitative real-time TaqMan assay as previously described (28, 29). Because the candidate regulatory variant resides within a non-coding region a common C/A SNP, rs1044059, in the 5’ UTR of RNASET2 that is in strong LD (R2 = 0.99) with tagging SNP rs1819333 was used as a surrogate marker. Real-Time TaqMan assay for rs1044059 (Applied Biosystems, Carlsbad CA) was used with the VIC-TaqMan probe detecting the A, non-risk allele, and FAM-TaqMan probe detecting the C, risk allele transcript. Sequential dilution of samples from healthy subjects homozygous for either the A-allele or C-allele were used to validate the assay for probe efficiency (**Supplemental Figure S7**). The relative expression of RNASET2 was normalized to ACTB as a reference gene using the following primers-probe set: Fwd: 5’-CGTGTGGCTCCCGAGGAG-3’; ACTB Rev: 5’-GGATAGCACAGCCTGGATAGC-3’; ACTB probe: 56-FAM/CCCCGTGCTGCTGACCGAGGC/3BHQ1. Allelic expression was evaluated by determining the ratio of transcript levels for each allele/total expression.

### Generation of promoter-reporter constructs

RNASET2 promoter regions were PCR amplified from genomic DNA isolated from individuals identified as risk and non-risk for rs2149092. PCR products spanning -4kb and -3.6 kb were digested with KpNI and ligated into KpnI/EcoRV cut pGL4.23 luciferase reporter vector. Primers used for amplification were: - 4kb FWD 5’-TTTGGTACCAGTGGCATCTGACGCATAG-3’; -3.6kb FWD 5’-TTTGGTACCAGACCCAAATCCAGCCTT-3’; 162bp REV 5’-GACGGCCTAAACCAGTATCTC-3’. Single base substitution of rs2149092 risk/non-risk promoter constructs was generated using Quickchange Site-directed mutagenesis kit (Agilent La Jolla, CA) and were validated by sequencing. Substitution primers targeting the reverse strand were: risk to non-risk CAGTACAGGAaGAAGTGACAAGAATTC or non-risk to risk CAGTACAGGAgGAAGTGACAAGAATTC with a Common Reverse: CCCACTTCTACTCCCCCA oligonucleotide.

### Luciferase assay

PBMC cells were transfected using a technique modified from that described previously (30). Briefly, isolated cells were rested overnight then washed and resuspended in 250 µl fresh medium at 1 x 107 cells/ml and electroporated in the presence of 50 µg of reporter construct (600 V, for 9 pulses of 500 µsec, with 100 µsec between pulses) using 4 mm (gap width) cuvettes in a BTX Electro Square Porator ECM 830 (Genetronics, Inc., San Diego, CA). A control plasmid containing the β-actin promoter driving a Renilla luciferase (provided by Dr. Christopher Wilson, University of Washington) was co-transfected as an internal standard and values were normalized to correct for transfection efficiency. After electroporation, the cells were diluted in fresh medium, allowed to rest for 1 h prior to plating, and then stimulated with either 100ng/ml TL1A (Fitzgerald, Concord, MA) plus 0.5ng/ml IL12 (Peprotech Rocky Hill, NJ), 50ng/ml IL18 (MBL Woburn, MA) and IL15 20 ng/ml (Peprotech Rocky Hill, NJ), with 2.5ul/ml ImmunoCult human CD3/CD28/CD2 T cell activator (StemCell Technologies, Tustin, CA) for 4 h. Luminescence was measured using a Promega (Madison, WI) dual-luciferase assay kit and counted on the CLARIOstar microplate reader (BMG Labtech Cary, NC). Relative luciferase activity was quantified compared to empty pGL4.23 vector.

### 4-Thiouridine labeling and separation of nascent and pre-existing RNA

4SU metabolic labeling was performed as previously described (31). Briefly, 250uM 4SU were added to cultured PBMC 4h prior to RNA isolation. 4SU-labeled RNA was biotinylated using EZ-link Biotin-HPDP (Thermo Scientific- Pierce, Waltham, MA) followed by streptavidin binding using uMac Streptavidin kit (Miltenyi, Auburn, CA). Reaction was loaded onto uMac columns and labeled and unlabeled RNA fractions were collected separately. Both fractions were precipitated, cleaned, and resuspended for quantification of expression. The ratio between pre-existing (untagged) and nascent (tagged) RNASET2 and ACTB mRNA were normalized for cDNA input and calculated based on the respective differences in Ct value between pre-existing and nascent transcripts,

### Flow cytometry

CD4^+^ T cells were stimulated with IL12/IL18/IL15 and TL1A or were left untreated for 72hrs. Cells were fixed, permeabilized and stained sequentially for intracellular RNASET2 using Ms anti RNASET2 (H00008635-B01P Novus Biological) followed by Goat anti mouse Alexa-fluor 488 and then for IFN-γ (brilliant violet 421-Ms anti IFN-γ, eBioscience). Cells were acquired on a FACSsymphony cell analyzer (BD Biosciences, San Jose) and data was analyzed with FlowJo software (TreeStar Inc., Ashland, OR).

### RNASET2 overexpression

CD4^+^ T cells were transfected with 25ug of pQCXIP-RNASET2-HIS or pQCXIP control vector (both kindly gifted by Dr. Geng Wang from Tsinghua University) using electroporation protocol described above. Cells were rested overnight and then stimulated with TL1A/cytokine cocktail (100ng/ml TL1A, 0.5ng/ml IL12, 50ng/ml IL18 and 20ng/ml IL15) for 24h. RNASET2 overexpression was validated by western blot, ELISA and/or qPCR. For experiments using recombinant RNASET2, purified from the expression vector or commercial (Cat. 13509-H08B Thermo-Fisher Asheville NC), cells were pretreated for 24h with indicated concentration of recombinant protein prior to stimulation. Cell pellets were collected for RNA analysis and culture media for IFN−γ secretion by ELISA.

### Quantification of plasma RNASET2

To quantify circulating blood plasma levels of RNASET2, an ELISA was developed (**Supplemental Figure S6**) with detection range of 100pg/ml-2ng/ml (**Supplemental Figure S6D**). High binding ELISA plates were coated overnight with 50μl of 1/100 diluted Rabbit anti-RNASET2 (cat. 041159 US biological Salem, MA). Plates were washed and samples and standards were added for 24h followed by addition of 50ul of 1/300 diluted polyclonal mouse anti-RNASET2 (cat. H00008635-B01P Novus Biological Littleton, CO) for 2h. This was followed by washing and addition of 100μl of 1/2000 diluted alkaline phosphatase-conjugated goat anti mouse (Jackson ImmunoResearch Laboratories, West Grove, PA) for 30 min. Substrate, 0.2 mM NADP (Sigma-Aldrich, St. Louis, MO) was added for 30 min followed by addition of amplifier (3% 2-propanol, 1 mM iodonitrotetrazolium violet, 75μg/ml alcohol dehydrogenase, and 50μg/ml diaphorase; Sigma-Aldrich) for 10 min. Plates were read at 490 nm using an E max plate reader (Molecular Devices, Sunnyvale, CA).

### Chromatin functional annotations

Chromatin states were annotated using StatepaintR (http://statehub.org/) based upon available Roadmap Epigenomics marks and ETS binding region using FANTOM (functional annotation of the mailman genome, https://fantom.gsc.riken.jp/data/) web tools. DNA topography was analyzed using the DNAshape and TFBSshape (https://rohslab.usc.edu/tools.html) bioinformatic tools. Regulatory Sequence Analysis Tool (RSAT) was applied for screening for predicted motif disruption of transcription factor (TF) binding sites using the JASPAR motif collection (32). Only T cell specific TFs (33 TFs) identified using RNAseq data from CD patients as being expressed, were carried forward. Postar3 platform was used for screening for RNA-binding protein mediated post-transcriptional regulation (33).

### Statistical analysis

Tests for statistical significance were determined using JMP Statistical Software (Cary, NC). Data were assessed for normality by the Shapiro-Wilk test. If data were normal a 2-tailed, unpaired Student’s t test was used. For non-normal data, Wilcoxon Test was used to calculate P values. Quantification for significance when evaluating expression of nascent *vs.* pre-existing RNASET2 transcripts was performed by comparing paired response to TL1A stimulation, prior and post 4SU-tagging, in samples isolated from same individual. Analysis for identifying alteration in circulating RNASET2 protein levels was performed by comparing paired sample RNASET2 protein levels prior and post-surgery for same individual patients. Paired sample significance was calculated using Wilcoxon signed rank test.

## Results

### RNASET2 single nucleotide polymorphism, rs2149092, is associated with distinctive T cell chromatin states and structural DNA features

**Figure 1:**
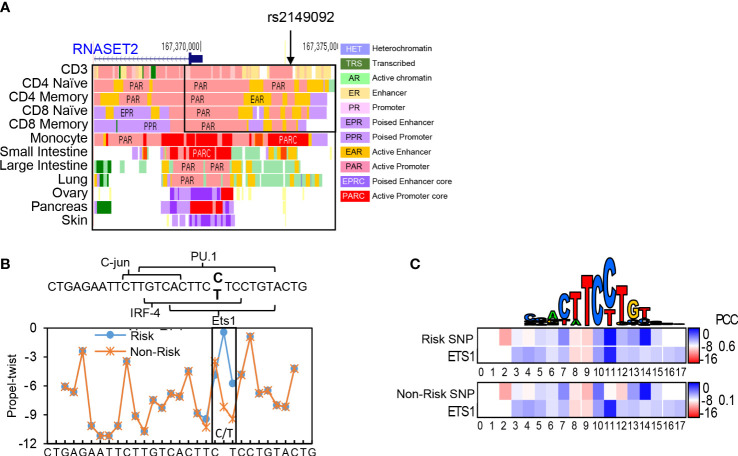
Cis-Regulatory SNP, rs2149092, on RNASET2 promoter is predicted to alter ETS TF binding. **(A)** StateHub modeling of RNASET2 in proximity of SNP rs12149092 **(B)** DNA topography analysis demonstrating that SNP rs2149092 (C>T) is predicted to alter DNA propeller twist configuration at the ETS1 binding site. **(C)** Heat map and Pearson’s correlation coefficients (PCC) comparing region containing rs2149092 C and T SNP to consensus ETS1 binding site.

Previously, we characterized an association of the rs1819333 RNASET2 disease risk variant with decreased T cell expression, DNA hypermethylation and clinical parameters of severity in CD patients (27). A putative regulatory variant, rs2149092, in linkage disequilibrium (LD) with the CD disease risk tagging SNP, was predicted to alter a consensus E26 transformation specific (ETS) transcription factor binding site. The rs2149092 SNP lies within an enhancer region overlapping with IRF4 and SPI.1 and directly adjacent to C-jun binding sites, suggesting the likelihood of a multifaceted cis-regulatory element impacting protein-DNA complex formation. Considering that epigenetic and environmental signals play a critical role in regulating enhancer activity and gene expression, it is essential that studies are performed in the cell type relevant to disease. The impact of rs2149092 in the context of T cell transcriptional regulation was examined for the presence of tissue-specific active enhancer/promoter regions using StatepaintR (34). As seen in **Figure 1A**, rs2149092 is located within active T cell enhancer (yellow) and promoter (pink) regions. A similar pattern is seen for CD3^+^, CD4^+^ or CD8^+^ naive and memory T cells. However, enhancer regions were depleted when comparing the chromatin state in other tissues including monocytes and large *vs.* small intestine suggesting allele specific as well as tissue-specific transcriptional regulation. Studies have shown that transcriptional regulation *via* protein-DNA binding depends on recognition of the three-dimensional DNA structure and that SNP sequences surrounding core TF binding motifs can modify DNA conformation resulting in altered TF binding activity (35, 36). We used the bioinformatic tools DNAshape and TFBSshape to evaluate the impact of the rs2149092 C/T SNP on DNA topography and the consequence on DNA-binding of ETS, IRF4 and SPI.1 proteins to this site. As seen in **Figure 1B**, the C/T SNP is expected to distort the “propeller twist three-dimensional” shape of DNA (boxed area), which is predicted to impact the core TF-DNA interaction with ETS (heat map and PCC when compared to consensus ETS1 binding site, **Figure 1C**) but not the binding of IRF4 and SPI.1 (data not shown).

### RNASET2 disease risk variant rs2149092 is associated with allele-specific ETS complex binding to promoter regulatory region

**Figure 2:**
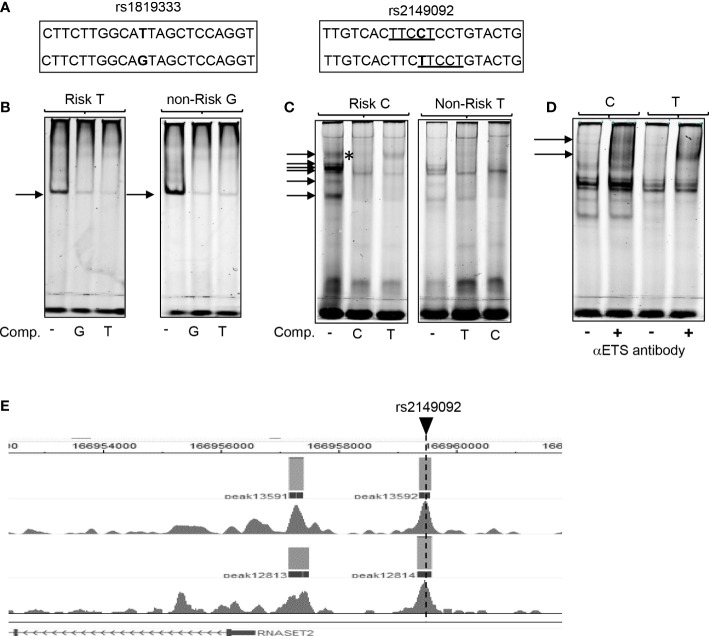
Allele specific nucleo-protein binding to RNASET2 promoter regions. **(A)** Sequence of rs1819333 and rs2149092 oligonucleotide probes. ETS1 TFBS are underlined. **(B–D)** EMSA analysis using nuclear extract from CD4^+^ T cells binding to **(B)** rs1819333 **(C)** rs2149092 or **(D)** rs2149092 supershifted complexes in the presence of ETS1-specific antibody. **(E)** ETS1 Chip-seq data from human purified T cells (FANTOM project). Unlabeled oligo competitors (comp): rs2149092 C or T SNP. Representative of 2-4 experiments.

To assess the functional potential for rs2149092 to alter protein-DNA binding, EMSA analysis was performed using nuclear extracts isolated from primary CD4^+^ T cells and allele specific oligonucleotide probes (**Figure 2A**). There was no discernable difference noted in the nucleo-protein DNA complex binding to the risk *vs.* non-risk allele specific oligonucleotides corresponding to the rs1819333 tagging SNP (**Figure 2B**). Likewise, complex formation was disrupted *via* competition with either excess risk or non-risk unlabeled oligonucleotides supporting absence of rs1819333 allele-specific variant regulatory function. In contrast as seen in **Figure 2C** the binding patterns differed for rs2149092 C and T alleles with an additional DNA-protein complex formed with the C probe (**Figure 2C**, upper arrow). Competition with excess unlabeled oligonucleotides showed that the C, but not T, probe abolished C specific complex formation (C panel asterisk), indicating allele-specificity. Nucleo-protein binding to rs2149092 was supershifted by ETS1 specific monoclonal antibody. An additional C specific supershift complex (**Figure 2D**) was detected confirming an allele specific ETS1 component. Chip-seq data from human purified T cell validates binding of ETS1 to rs2149092 (**Figure 2E**).

### T cell stimulation attenuates RNASET2 expression

**Figure 3:**
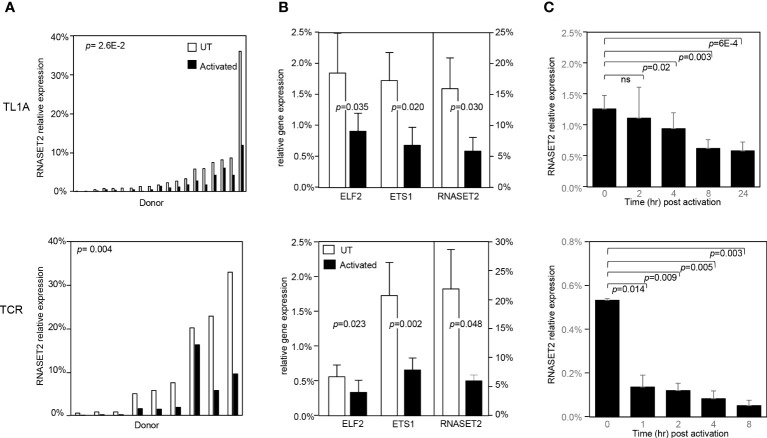
RNASET2 transcript downregulation is a hallmark of T cell activation. Expression of RNASET2 in CD4^+^ T cells activated with TL1A (upper panels) or TCR (lower panels). **(A)** Expression of RNASET2 is decreased in cells isolated from multiple donors (TL1A *n*=22, TCR *n*=9). **(B)** Concordant decrease in ELF2, ETS1 and RNASET2 (TL1A *n*=5, TCR *n*=6). Data show the mean ± SD. **(C)** Kinetics of RNASET2 expression following activation (TL1A *n*=8, TCR *n*=3). ns, not significant.

To address whether a decrease in RNASET2 expression was specific to TL1A-mediated activation or more globally associated with other classic modalities of T cell activation, such as T cell receptor (TCR) or PMA/Ionomycin, CD4^+^ T cells were stimulated with the different activating agents and RNASET2 expression was measured. Activation of T cells *via* TL1A as well as TCR or PMA/ionomycin resulted in down regulation of RNASET2 expression (**Figure 3A** and **Supplemental Figure S1A**). A 40- 50% decrease was observed following TL1A stimulation while a 75-90% decrease was observed following TCR or PMA/ionomycin activation. The expression of ETS transcription factor family members ETS1 and ELF2 was likewise evaluated. As seen in **Figure 3B**, both TL1A and TCR stimuli elicited a parallel downmodulation of RNASET2, ETS1 and ELF2 further supporting a role for ETS in regulating RNASET2 expression. The kinetics of downregulation following TL1A or TCR activation (**Figure 3C**) revealed that while TL1A induced a gradual decline, with the half-life of RNASET2 transcript calculated to be 8 hours, TCR stimulations resulted in a rapid decrease with a half-life of 0.8 hours following activation (**Supplemental Figure S1B**).

### RNASET2 disease risk variants are associated with allele-specific promoter transactivation

**Figure 4:**
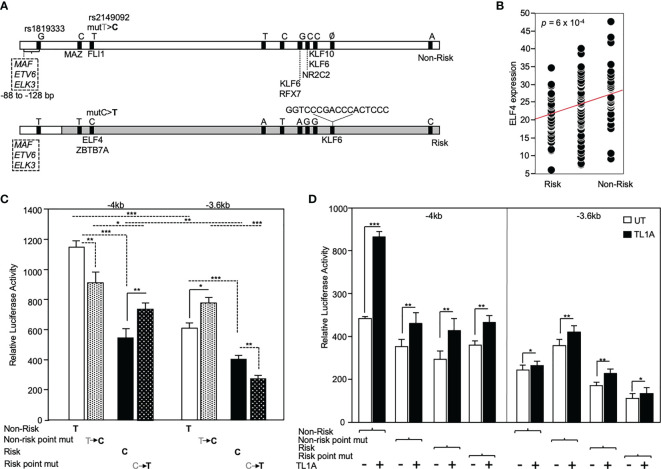
Functional analysis of RNASET2 promoter SNP variants. **(A)** Schematic illustration of luciferase reporter construct used to analyze functional contribution of risk and non-risk index rs1819333 and regulatory rs2149092 variants on RNASET2 promoter activity. Tick marks represent SNPs in LD with both rs1819333 tagging and rs2149092 regulatory variants. Transcription factors (TF) associated with both a putative SNP-dependent binding site and with RNASET2 eQTL risk carriage are listed. Boxed region directly upstream of rs1819333 depicts TF binding sites for ETS family transcriptional repressors. **(B)** EQTL ELF4 expression *vs.* rs1819333 allelic carriage. **(C, D)**. CD4^+^ T cells were transfected with promoter-reporter vectors. **(C)** Resting (-4kb, n=16, -3.6kb, *n*=22) or **(D)** TL1A (-4kb, *n*=8, -3.6kb *n=8*) activated cells. Data show the mean ± SD ***P < 0.001, **P < 0.01; *P < 0.05.

The functional significance of rs2149092 regulatory SNP on RNASET2 gene transcription was examined utilizing promoter reporter constructs. A -4kb DNA construct was cloned from individuals homozygous for both the risk and non-risk rs2149092 regulatory and rs1819333 disease tagging SNPs and a truncated -3.6kb construct was generated defined by the presence rs2149092 SNP. Additionally, a single base pair substitution of the rs2149092 non-risk T to C as well as risk C to T were introduced within the -4kb and -3.6 kb constructs (**Figure 4A**). Sequencing the promoter revealed the presence of 8 additional variants with strong association (R2>0.8) to both rs2149092 and rs1819333 SNPs. SNP-dependent putative transcription factor binding sites were identified by motif analysis. Thirty one TF factors were identified (**Supplemental Table 1**) of which 50% have been associated with differential gene expression in whole blood from CD compared to non-IBD subjects (37). All TFs were analyzed for trans eQTL association of expression with RNASET2 disease risk genetic variation carriage. Expression of eight transcription factors were significantly associated with rs1819333/rs2149092 disease risk carriage (**Figures 4A, B** and **Supplemental Table 1**).

Across all promoter regions the non-risk variant demonstrated enhanced promoter activity as compared to risk (**Figure 4C**). Within the context of the -4kb promoter region, spanning both rs2149092 and rs1819333 variants, a single base pair substitution of the rs2149092 non-risk T to risk C, was associated with a significant decrease in promoter activity whereas substitution of risk to non-risk enhanced promoter activity. However, in the absence of the rs1819333 tagging SNP (-3.6 kb construct), substitution of the rs2149092 non-risk T to risk C was associated with increased promoter activity whereas substitution of the risk to non-risk decreased promoter activity (**Figure 4C**). These finding suggest that promoter regulation is dependent upon the cooperative interaction of multiple cis- and trans-regulatory elements which play a critical role in selective transcription factor binding and gene activation. Indeed, motif analysis identified multiple ETS factor transcriptional repressor binding sites within the -4kb region directly upstream of rs1819333 (**Figure 4A** boxed region). Surprisingly, although T cell activation results in an overall decrease in RNASET2 mRNA levels (**Figure 3**), TL1A (**Figure 4C**) stimulation enhanced RNASET2 promoter activity in all reporter constructs.

**Figure 5:**
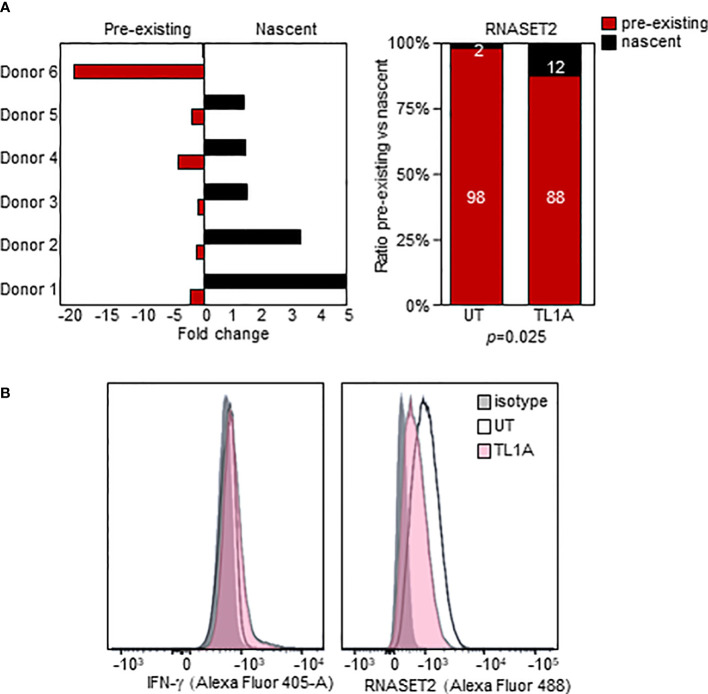
TL1A activation inversely impacts nascent and pre-existing RNASET2 transcripts. **(A)** Increase in nascent (*p*=0.047) and decease in pre-existing (*p*=0.03) RNASET2 transcript from PBMC cells treated with TL1A. Levels are plotted as fold over expression in untreated cells (*n*=6). **(B)** Intracellular IFN-γ protein increases and RNASET2 protein decreases following TL1A stimulation. Flow cytometry analysis representative of three with similar results.

We used an *in-vitro* metabolic RNA labeling approach to further investigate the role of transcriptional upregulation v. mRNA stabilization as mechanisms involved in TL1A mediated regulation of RNASET2 expression. PBMC were activated with TL1A and then cells were labeled using 4-thiouridine (4sU) incorporation, a naturally occurring uridine analog to distinguish between nascent and previously transcribed mRNA populations. As seen in **Figure 5A**, while levels of previously transcribed RNASET2 mRNA decrease following TL1A activation, an increase in newly transcribed mRNA was detected, reflecting the results of RNASET2 promoter activity observed using reporter constructs. No alteration between nascent and previously transcribed beta actin, following TL1A stimulation, was detected (**Supplemental Figure S2**). Nevertheless, intracellular expression of RNASET2 protein is decreased in response to TL1A stimulation and a concomitant increase in intracellular IFN-γ protein was observed (**Figure 5B**).

### RNASET2 disease-risk variant dominates allele-specific expression

**Figure 6:**
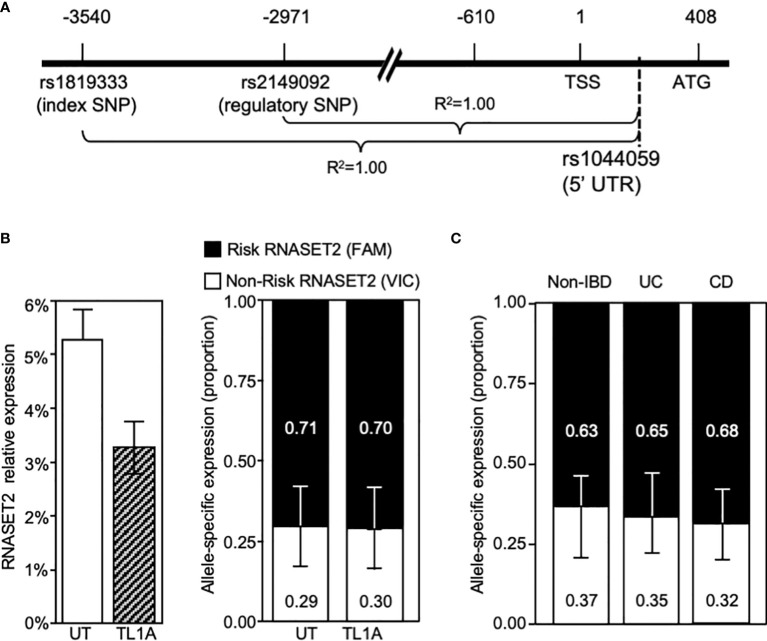
RNASET2 Risk variant dominates RNASET2 expression. **(A)** Diagram mapping the location and LD of rs1044059 on RNASET2 promoter. **(B)** Analysis in CD4^+^ T cells of total RNASET2 expression (left panel) or allele specific expression (right panel) with or without TL1A activation of (n=12). Allele specific expression in **(C)**. Resting PBMC isolated from non-IBD (*n*=6), CD (*n*=9) or UC (*n*=8) subjects. Across all samples the allele specific ratio of risk *vs.* non-risk expression was ~ 2:1 with a *p* value <0.001.

The disconnect between RNASET2 mRNA levels and promoter activity following TL1A activation suggests that transcriptional as well as post-transcriptional regulatory mechanisms may be involved in modulating the levels of RNASET2 expression. Allele-specific expression (ASE) was examined to determine the functionality of the risk variants and whether there is an imbalance between the expression levels of the risk *vs.* non-risk alleles. Because our index and regulatory variants reside within a non-coding region we identified a common C/A SNP, rs1044059, in the 5’ UTR of RNASET2 that is in strong LD (R2 = 0.99) with the index and regulatory SNP as a surrogate marker (**Figure 6A**). CD4^+^ T cells isolated from healthy donors (heterozygous for rs1819333) were treated with or without TL1A or TCR stimulation. Expression of the two alleles were assayed in a multiplex reaction using an adaptation of TaqMan SNP genotyping assay as previously described (31). Considering that the risk allele is associated with a decrease in RNASET2 expression we anticipated that the risk *vs.* non-risk allele-specific expression would deviate from a 50/50 ratio. As seen in **Figure 6B** right panel, in unstimulated resting T cells an allelic imbalance in expression was indeed detected. Paradoxically the allelic-imbalance was driven by the risk rather than the non-risk allele, with expression of the risk allele being twice as high as non-risk (ratio 2:1). Moreover, despite an overall decrease in the levels of total RNASET2 mRNA expression following TL1A or TCR cell activation (**Figure 6B** left panel and **Supplemental Figure S3**), the relative ratio of each allele was maintained with the risk allele driving overall expression.

Allelic imbalance in expression was likewise examined in primary cells in the context of disease (**Figure 6C**). PBMC were collected from CD and ulcerative colitis (UC) patients undergoing surgery for disease management and non-IBD subjects. A similar pattern of risk allele expression dominancy was detected in all subjects. The data utilizing promoter reporter constructs and allele-specific RNA expression analysis suggest that both allele-specific cis-regulatory as well as allele-specific post-transcriptional mechanism may ultimately determine the level of RNASET2 expression.

### RNASET2 disease risk variant is associated with decreased circulating protein

**Figure 7:**
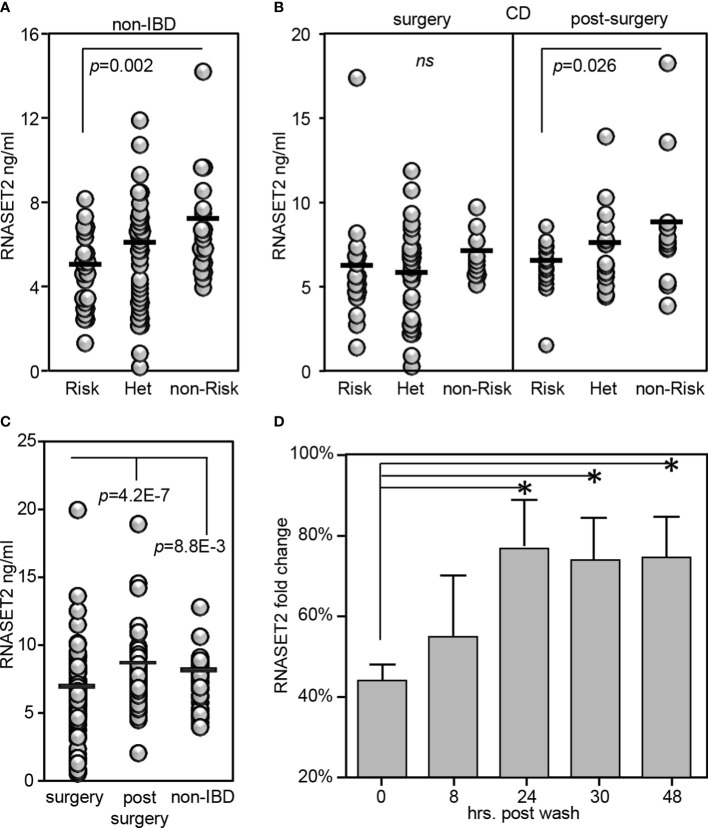
RNASET2 risk variant is associated with decreased levels of circulating protein. Circulating RNASET2 protein levels associated with allelic carriage in **(A)** non-IBD subjects (*n*=84) or **(B)** CD patients at time of surgery (*n*=93 left panel) and up to one year post surgery (*n*=47 right panel). **(C)** Increase in circulating levels of RNASET2 following surgical removal of inflamed intestinal region. **(D)** Recovery of RNASET2 transcript levels in CD4^+^ T cells following removal of TL1A activation (*n*=6). Data show the mean ± SD. *P < 0.05.

RNASET2 is a secreted protein involved in macrophage chemotaxis and tissue remodeling and is induced following exposure to stress (38). Thus, the level of circulating protein may well provide further insight into the functional relevance of allelic variant carriage. We developed an ELISA assay to evaluate the plasma levels of circulating RNASET2 protein in the context of risk allele carriage and in the context of disease. In healthy control subjects, the levels of circulating RNASET2 echoed mRNA expression and was associated with SNP variant carriage with the levels of protein significantly decreased in subjects homozygous for the RNASET2 risk allele. (**Figure 7A**). To evaluate RNASET2 levels in disease, blood samples were collected from CD patients with medically-refractory disease requiring surgical intervention for disease management. The levels of RNASET2 in samples collected from CD patients at time of surgery was significantly decreased compared to healthy subjects and there was no association with disease risk variant carriage (**Figure 7B**, left panel). However, within a year following surgery the levels of circulating RNASET2 in most patients (60%), irrespective to allelic carriage, increased to levels comparable to those of healthy subjects (**Figure 7B**, right panel and **7C** and **Supplemental Figure S4**). Likewise, circulating RNASET2 protein was now associated with SNP variant carriage with the levels significantly decreased in CD patients homozygous for the RNASET2 risk allele (**Figure 7B**, right panel). CD patient longitudinal follow-up samples, collected up to 3 years post-surgery, revealed that the post-operative increase in levels of circulating RNASET2 were maintained (**Supplemental Figure S4**). These data suggest that removal of the inflammatory triggering source *via* surgery may reset the level of circulating RNASET2 protein. To further test this hypothesis CD4^+^ T cells were activated *via* TL1A for 24 hours followed by a washout of TL1A, and RNASET2 transcript levels were measured. As shown in **Figure 7D**, within 24 hours following washout, RNASET2 transcript recovered to 75% of its level prior to TL1A stimulation.

### Recombinant RNASET2 inhibited TL1A-mediated IFN-γ secretion

**Figure 8:**
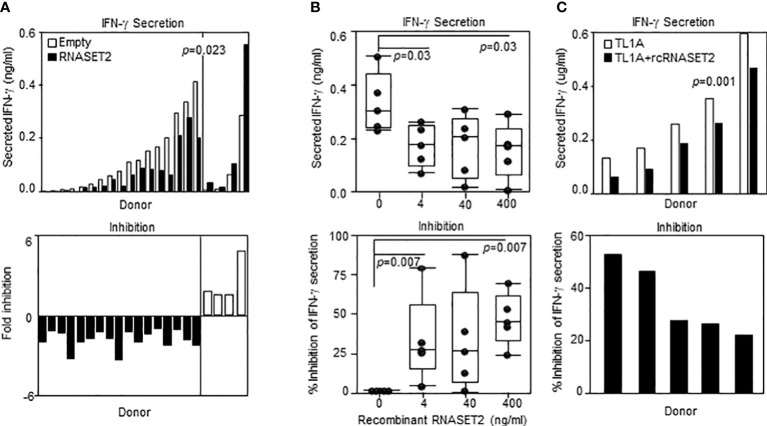
Recombinant RNASET2 attenuates IFN-γ secretion in a dose dependent manner. CD4^+^ T cells were either **(A)** Transfected with RNASET2 overexpression vector (*n*=21) or **(B)** Treated with recombinant RNASET2 prior to TL1A stimulation (*n*=5). **(C)** Resting PBMC isolated from IBD subjects (CD *n*=2; UC *n*=3) treated with 400 ng/ml recombinant RNASET2 protein. IFN−γ secretion was measured after 24 hours. Bottom panels depict fold inhibition in IFN−γ secretion comparing overexpression *vs.* empty vector or recombinant RNASET2 treatment *vs.* no recombinant protein.

Our results show that decreased levels of RNASET2 is associated with disease and inflammatory cytokine secretion and that downregulation of RNASET2 is reversible following removal of inflammatory signal. Thus, RNASET2 may represent a potential novel therapeutic target in the treatment of IBD. We therefore proceeded to investigate whether raising the levels of RNASET2 had a direct effect on attenuation of cytokine secretion. CD4^+^ T cells, isolated from healthy donors, were transfected with an RNASET2 over-expression or empty vector and the levels of secreted IFN−γ were measured following TL1A activation. As seen in **Figure 8A**, in the majority (16/21) of the donors, over-expression of RNASET2 resulted in a significant decrease in TL1A mediated IFN−γ secretion (average 40% decrease, p=0.023). Donor attenuation of IFN−γ secretion in response to elevated levels of RNASET2 was consistent and reproduceable. Cells isolated from the same donor up to 2 years later displayed similar results (**Supplemental Figure S5**). The potential of recombinant RNASET2 protein (recRNASET2) to directly modify the inflammatory response was likewise investigated. CD4^+^ T cells were pre-treated with or without recRNASET2 prior to TL1A activation and IFN−γ secretion levels were evaluated. As seen in **Figure 8B** pretreatment with recRNASET2 resulted in attenuation of IFN−γ secretion in a dose dependent manner. As little as 4ng/ml of recRNASET2 induced a 30% reduction in IFN−γ secretion with a further decrease in IFN−γ secretion (45%) in the presence of 400ng/ml recRNASET2. Likewise, recRNASET2 mediated attenuation of IFN−γ secretion was observed in cells isolated from IBD patients (**Figure 8C**).

## Discussion

RNASET2 has been reported to play multiple roles exerting both pro- inflammatory and anti-inflammatory roles in disease. In the context of ovarian cancer and lymphoma, RNASET2 functions as an oncosuppresor (39–41). Secretion of RNASET2 in the tumor environment defines its role as a potential ‘alarmin’ galvanizing an innate immune response and recruitment of M1-polarized macrophages (17). Additionally, it plays as essential for TLR8 activation on macrophages in response to intracellular pathogens and subsequent TH1 cell activation (14, 15). Recent studies in ovarian cancer models suggest that RNASET2 contributes to maintaining the balance of M1/M2 macrophages in the tumor environment and may impact upon T cell adaptive immune memory response (19, 42). Likewise, Rnaset2 −/− mice develop not only neuroinflammatory encephalopathy but autoinflammatory organ disease and a disrupted hematopoiesis as well (43). In contrast, enhanced levels of RNASET2 is are detected in vitiligo patient specimens and can be induced *in vitro* in cultured primary human melanocytes and keratinocyte in response to stress (44). We have previously reported that downregulation in RNASET2 expression is a component of TL1A-mediated enhancement of IFN−γ secretion and in CD patients the RNASET2 disease risk variant is associated with decreased expression and increased IFN−γ expression (27). There is a functional association in IBD patients between DNA hyper-methylation and decreased expression of RNASET2, and a significant eQTL overlap with RNASET2 rs1819333 IBD-risk variant carriage. Moreover, RNASET2 disease-risk SNPs were associated with therapeutic failure of anti-TNF therapy, penetrating disease, a history of multiple resections with a shorter time to repeat surgery all of which are clinical characteristic of overall disease severity. TF motif analysis across all variants demonstrating eQTL and mQTL prospectively identified a regulatory SNP, rs2149092, predicted to alter a conserved ETS consensus binding sequence, adjacent to composite overlapping TF binding sites, suggesting combinatorial transcriptional gene regulation of expression. In this study we used an integrative multi-strategy approach to further identify cis- and trans-regulatory mechanisms mediating downregulation of RNASET2 expression and enhancement of pro-inflammatory cytokine expression/secretion. Our main findings demonstrate: 1) Allele specific cis-regulatory binding motifs associated with active chromatin regulatory features and ETS1 nucleo-protein complex; 2) Allelic imbalance in RNASET2 promoter transactivation and mRNA levels supporting multilevel transcriptional and post-transcriptional regulatory mechanism complexity modulating RNASET2 expression; 3) Pre-operative decrease and post-operatively recovery in circulating RNASET2 protein levels in CD patients requiring surgical intervention for disease management following removal of the inflamed intestinal region; and 4) Biological activity of recombinant RNASET2 protein in attenuating pro-inflammatory cytokine secretion.

In further defining the RNASET2 cis-regulatory features of rs2149092 we determined that the chromatin state of rs2149092 was enriched for epigenomic marks indicative of T-cell specific active enhancer and promoter regulatory elements. RNASET2 variant TF–DNA binding in the context of cell and tissue-specificity is of importance considering tissue-specific functionality related to altered RNASET2 expression. For example, in the case of activated T cells, a decrease in RNASET2 expression is associated with enhanced IFN-γ secretion whereas in activated granulocytes an increase in RNASET2 expression is associated with triggering innate immune response and recruitment of macrophages (18, 45). DNA topography analysis integrating the DNA shape and sequence features indicated that SNP rs2149092 altered the 3D DNA conformation at an ETS TF binding site. EMSA nucleo-protein/super-shift data corroborated the potential for RNASET2 regulatory SNP rs2149092 to modulate ETS TF DNA interactions. In this context, it is interesting to note a recent study in which the DNA shape was reported as an important contributing factor to ETS TF mediated transcriptional regulation (46). A parallel downmodulation of RNASET2, ETS1 and ELF2, following T cell activation strengthens a role for ETS in regulating RNASET2 transcription. In contrast, no allele-specificity was observed in nucleo-protein complex formation to the RNASET2 tagging SNP rs1819333. ETS TF is of particular relevance to T cell biology implicated in T cells differentiation, pro-inflammatory cytokine/chemokine expression and downstream signaling pathways (47, 48). Numerous studies support a role for ETS TF regulation in IBD. ETS TF binding sites were enriched within IBD susceptibility enhancer and promoter regions (48, 49) and ETS TF risk variants themselves were associated with IBD (50–52). Clinically, elevated expression of ETS TF was observed in inflamed mucosa from IBD patients and was associated with enhanced pro-inflammatory cytokine secretion (53) and fistulizing disease (54). These data highlight ETS involvement in IBD pathogenesis.

To gain insight into the functional consequence of RNASET2 rs2149092 SNP on gene expression, we utilized promoter reporter constructs isolated from individuals homozygous for the risk or non-risk variant within context or independent of the rs1819333 tagging SNP. Eight additional variants were identified in LD with rs1819333/rs2149092 SNPs. Motif analysis was used to predict allele-specific binding. Of the TFs predicted to display allele-specific binding, **~** 30% displayed trans-eQTL with gene expression significantly associated with rs1819333/rs2149092 disease risk carriage and virtually all have previously been described as transcriptional regulators in IBD (55–61). Moreover, a recent single-cell RNAseq study integrating cell-specific-eQTL to RNASET2 disease risk variants provides strong evidence that multiple immune cell subsets including CD4+ T cells contribute to Crohn’s disease causation (62).

Our data reveal a multifaceted functional allelic imbalance in promoter transactivation. We detected decreased RNASET2 promoter activity when comparing the disease risk *vs.* non-risk alleles within context or independent of the rs1819333 tagging SNP. Within the context of the rs1819333 variant promoter region, a single base pair substitution of the rs2149092 non-risk T to risk C, down regulated promoter activity whereas a risk to non-risk substitution enhanced promoter activity. However, in the absence of the rs1819333 tagging SNP, the functional consequence is reversed. Substitution of the rs2149092 non-risk to risk increased promoter activity whereas the risk to non-risk swap decreased promoter activity. These findings suggest a regulatory role for rs2149092 dependent on the flanking sequence context. Indeed, the rs1819333 reporter construct includes a flanking region (-88 to -128bp) predicted to bind member of ETS family with transcriptional repressor activity which might partially explain this conundrum (63).

Surprisingly, while T-cell activation mediated a decrease in the overall levels of RNASET2 gene expression there was enhanced promoter transactivation for all promoter constructs following T cell activation. Promoter-reporter analysis is constrained to transcriptional regulation at a single point in time. It likewise does not measure accumulation of RNASET2 mRNA which is dependent on the balance between transcriptional upregulation *vs.* decay. In fact, 4sU-tagging of newly transcribed mRNA concurred with reporter assay results and demonstrated that following T cell stimulation there was a six-fold activation of the transcriptional machinery enhancing newly synthesized RNASET2 expression whereas, pre-existing RNASET2 transcripts underwent RNA degradation.

Allele specific expression (ASE) assay likewise demonstrated an allelic imbalance with risk allele dominance in gene expression in cells isolated from control subjects, as well as CD or UC patients. Allele dominance in gene expression of the risk allele compared to non-risk allele was observed in resting, as well as, activated cells. Considering our previous observation that RNASET2 disease risk variants are associated with decreased RNASET2 expression in both T cells and CD mucosal biopsies along with a more complicated/resistant CD phenotype the present results were rather unexpected. One possible explanation for these findings, might arise from differences in transcript levels and epistatic interactions in the presence of multiple cis-acting and/or trans-regulatory mechanisms. Our reporter assay supports the co-occurrence of multiple cooperative binding regions which likely contributes to haplotype-regulatory mechanisms. Comparison of RNASET2 levels between homozygous risk and non-risk individuals are subject to environmental differences in addition to cis- and trans-acting regulatory effects. In contrast allele specific expression is assayed from the two alleles from the same individual, which function as mutual internal controls, exposed to a shared cellular environment. Thus, differences between RNASET2 levels in individuals homozygous for risk *vs.* non-risk variants and those detected in heterozygote individuals could indicate that other trans-regulatory or cellular factors, apart from cis-acting elements on RNASET2 promoter, are involved in controlling the level of expression. Likewise, gene expression is regulated not only on a transcriptional level but by the rate of mRNA degradation. The promoter-reporter analysis measures transcriptional activation of potential core promoter regions while the allele specific expression adds to this information reflecting both transcriptional and post-transcriptional regulation. Posttranscriptional gene regulation by RNA binding proteins can significantly alter the stability of mRNAs. It is thus interesting to note recent studies suggesting RNASET2 post-transcriptional gene regulation demonstrating mRNA-destabilizing allele-specific RNA-protein binding associated with RNASET2 GWAS risk variants (64). Likewise aberrant RNASET mRNA splice variants were associated with a poor prognosis in renal carcinoma (65). Using the POSTAR3 database a number of predicted T cell post-transcriptional mRNA-destabilizing RNA-protein binding motifs have been identified for RNASET mRNA as well as N6-Methyladenosine and Nm (2’-O-methylation) mRNA modification believed to be a key mechanism affecting mRNA translation and stability. Moreover, RNASET2 protein has been reported to be targeted for FBXO6 unbiquitin-dependent protein degradation in ovarian cancer suggesting additional regulatory mechanism contribute to RNASET2 protein turnover (66). The present study will serve as a basis for future studies to unravel the disconnect between mRNA transcription and expression, as well as protein expression and define post-transcriptional mechanisms involved in these processes.

The clinical relevance of RNASET2 polymorphisms was further elucidated by our development of an RNASET2 protein ELISA. Changes in the levels of circulating RNASET2 were observed when comparing CD patients to healthy control subjects and in disease when comparing CD pre-operative to post-operative levels. Circulating RNASET2 protein levels in CD patients requiring surgical intervention for disease management, compared to healthy control subjects, was decreased. It is important to keep in mind that CD patients requiring surgical intervention have severe disease and usually have failed to achieve disease remission in response to medical therapies. Of note over 80% of these patients have failed to respond to therapeutic immunomodulation. Likewise, while in this patient population there was a significant decrease in circulating RNASET2 protein levels compared to healthy donor, there was a loss in association with disease risk variant carriage. Surgical resection of the inflamed tissue that is contributing to inflammation appears to restore a sustained increase in the levels of circulating RNASET2 and allelic association. Furthermore, overexpression or recombinant RNASET2 treatment attenuated pro-inflammatory IFN−γ secretion. While the source(s) of circulating RNASET2 protein remains to be determined these findings support potential for reversing an RNASET2 mediated inflammatory response *via* therapeutic enhancement of RNASET2 protein levels and continued studies utilizing *in-vivo* mouse models of chronic mucosal inflammation.

Taken together these data provide a solid foundation for further in-depth studies to evaluate the contribution of both allele-specific cis-regulatory as well as post-transcriptional trans-regulatory mechanisms in determining the overall level of RNASET2 expression. Additionally, our findings suggest that the combination of RNASET2 genetic markers and circulating protein levels may serve not only as a potential diagnostic tool in evaluating CD pathobiology but in addition help identify a subset of severe Crohn’s disease who may respond to RNASET2 as a novel therapeutic.

## Data availability statement

The data presented in the study are deposited in the GEO repository, accession number GSE215144.

## Ethics statement

The studies involving human participants were reviewed and approved by Cedars-Sinai Institutional Review Board. Written informed consent to participate in this study was provided by the participants’ legal guardian/next of kin.

## Author contributions

EB-R, ST, and RG contributed to conceptualization and study design. EB-R and FR designed experiments and carried out research. SC, DM, and DH acquired and performed data curation and SC and DH data analysis. EB-R drafted the article. ST and RG wrote the manuscript. DM, RG, and ST were responsible for funding acquisition. All authors critically reviewed the manuscript. All authors contributed to the article and approved the submitted version.

## Funding

This work was supported by the F. Widjaja Foundation (ST and RG) and the National Institute of Diabetes Digestive & Kidney Diseases (RO1 DK117893).

## Acknowledgments

We are thankful to all clinicians, coordinators and especially patients who have contributed time, data, and samples to the MIRIAD Biobank. The authors are grateful to Frank Kim for providing technical assistance.

## Conflict of interest

ST – is a stockholder and a consultant for Prometheus Biosciences; DM - is a stockholder and a consultant for Prometheus Biosciences Cedars-Sinai - has financial interests in Prometheus Biosciences, Inc., a company which has access to the data and specimens in Cedars-Sinai’s MIRIAD Biobank (including the data and specimens used in this study) and seeks to develop commercial products.

The remaining authors declare that the research was conducted in the absence of any commercial or financial relationships that could be construed as a potential conflict of interest.

## Publisher’s note

All claims expressed in this article are solely those of the authors and do not necessarily represent those of their affiliated organizations, or those of the publisher, the editors and the reviewers. Any product that may be evaluated in this article, or claim that may be made by its manufacturer, is not guaranteed or endorsed by the publisher.
