# Supplementary material for: Diagnostic and therapeutic potential of RNASET2 in Crohn’s disease: Disease-risk polymorphism modulates allelic-imbalance in expression and circulating protein levels and recombinant-RNASET2 attenuates pro-inflammatory cytokine secretion
Source: Front Immunol. 2022 Nov 16;13:999155. doi: 10.3389/fimmu.2022.999155 (PMC9709281; doi:10.3389/fimmu.2022.999155)
Supplement: Supplementary file 1 [file Image_1.pdf]

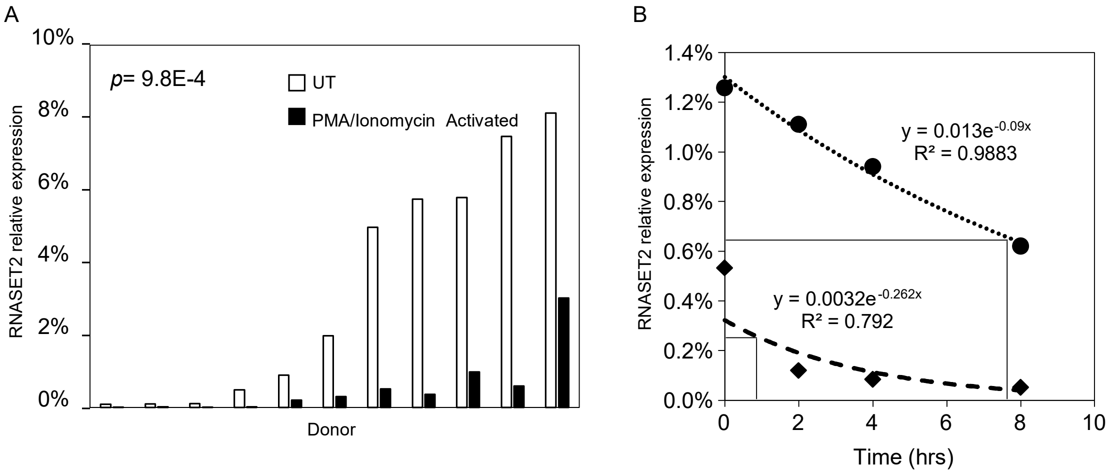

Supplementary figure S1. A. Decrease in expression of RNASET2 in cells isolated from multiple donors following PMA/ionomycin activation (*n*=11. B. RNASET2 mRNA decay following TL1A (circles) and TCR (diamonds) activation. Half life of RNASET2 mRNA is indicated on each tradeline (TL1A n=8, TCR n=3).

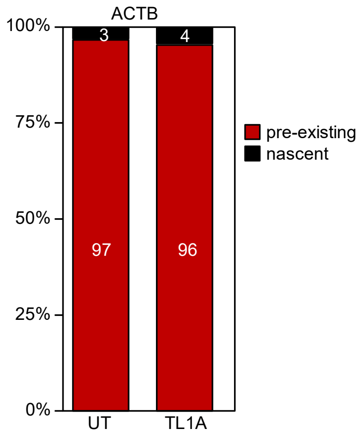

Supplementary Fig. S2. The ratio of pre-existing vs nascent ActB mRNA were unchanged following TL1A stimulation.

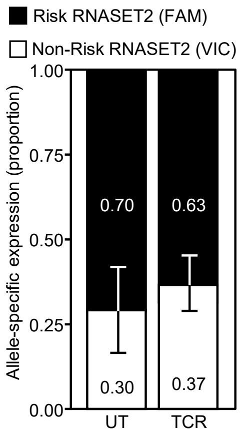

Supplementary figure S3. RNASET2 allele specific expression with or without TCR activation of (*n*=6). Data show the mean ± SD.

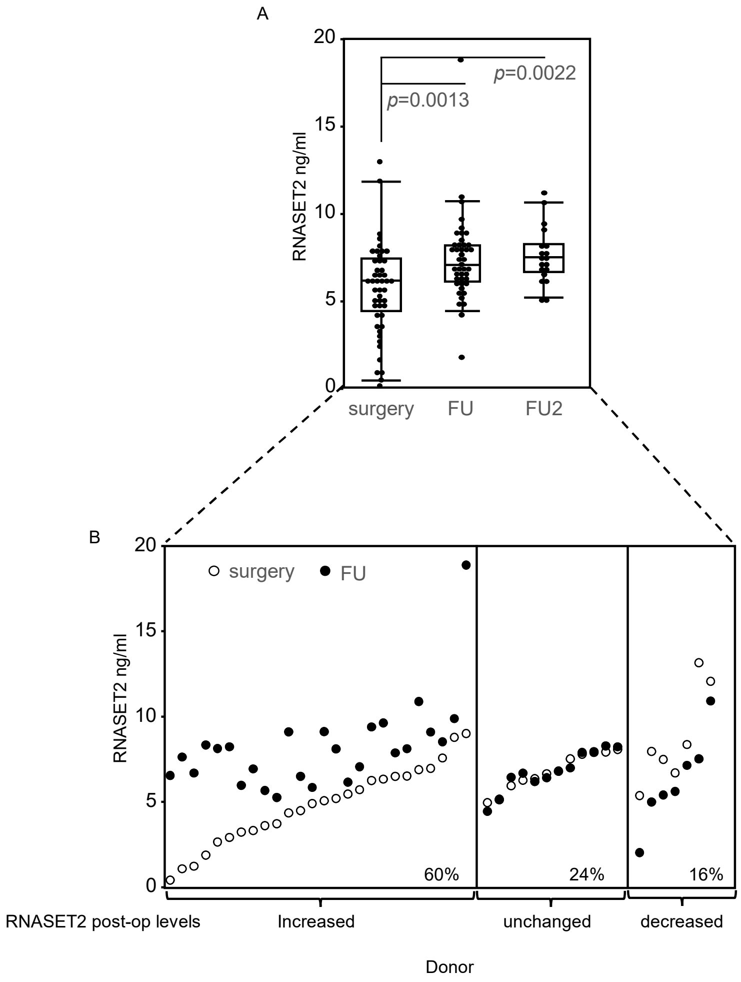

Supplementary Figure S4: A. Circulating RNASET2 protein levels from CD patients at time of surgery (*n*=98 left bar), up to one year (*n*=78 FU) and three years post surgery (*n*=31 FU2). B. Change in circulating RNASET2 protein levels comparing paired samples from same individual pre- and post surgery. Note that increase is observed in majority of patients.

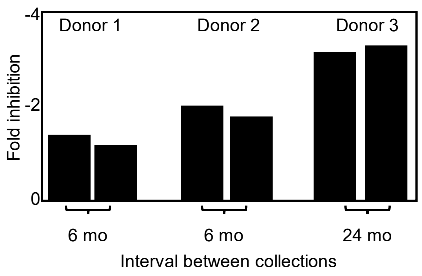

Supplementary Figure S5 : Overexpression of RNASET2 consistently attenuates IFN-γ secretion in same individual. CD4+ T cells, isolated from the same individual from collections 6-24 months apart, were transfected with RNASET2 overexpression vector prior to TL1A stimulation. IFN-γ secretion was measured by ELISA. Fold inhibition in IFN-γ secretion was measured by comparing levels in cells transfected with RNASET2 overexpression vs empty vector.

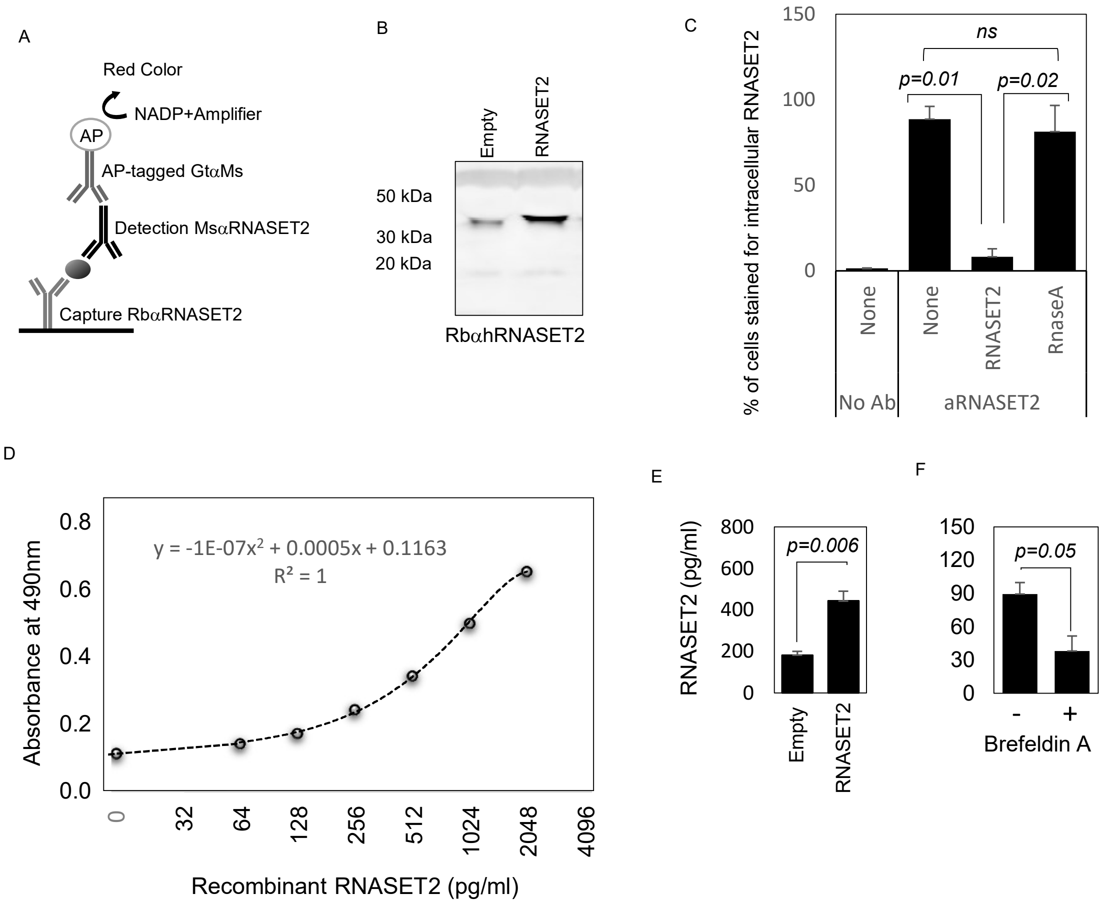

Supplementary Figure S6: Assessing sensitivity and specificity of RNASET2 antibodies used in RNASET2 ELISA. A. Schematic of RNASET2 ELISA (Rb-Rabbit, Ms-Mouse, Gt-Goat, AP-Alkaline phosphatase). B. Western blot assay confirming RNASET2 ELISA capture antibody capability to detect RNASET2 in cells transfected with RNASET2 expression or empty vector. Band on the left indicates detection of endogenous RNASET2. C. Flow cytometry assay of intracellular RNASET2 in T cells validates specificity of RNASET2 ELISA detection antibody after preincubation of antibody with competing excess (2.5ug/ml) recombinant RNASET2, RnaseA or no additional protein (none) (*n*=2). Data show the mean ± SD. Note that only recombinant RNASET2 but not RnaseA inhibited intracellular RNASET2 detection. D. Sensitivity and standard curve of RNASET2 ELISA (E,F). RNASET2 secretion measured by ELISA from T cells transfected with RNASET2 expression or empty vector (representative of 5 with similar results) (E) or treated with secretion inhibitor Brefeldin A (n=2) (F). Data show the mean ± SD.

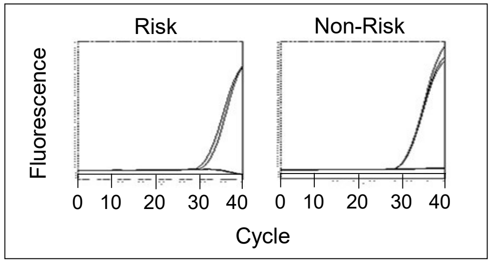

	FAM (Risk)	VIC (Non-Risk)
**Risk**	28.31	+40
**Risk:Non-Risk 3:1**	26.78	28.2
**Risk:Non-Risk 2:2**	27.25	27.82
**Risk:Non-Risk 1:3**	27.68	26.93
**Non-Risk**	+40	28.18

Supplementary Figure S7: **Validating allele specific RNASET2 expression assay**. Ct charts of samples from healthy subjects homozygous for risk and non-risk (top graphs) and Ct counts of sequential dilution of risk and non-risk samples confirming similar allele specific probes efficiency (table below).
