# Supplementary material for: Diagnostic and therapeutic potential of RNASET2 in Crohn’s disease: Disease-risk polymorphism modulates allelic-imbalance in expression and circulating protein levels and recombinant-RNASET2 attenuates pro-inflammatory cytokine secretion
Source: Front Immunol. 2022 Nov 16;13:999155. doi: 10.3389/fimmu.2022.999155 (PMC9709281; doi:10.3389/fimmu.2022.999155)
Supplement: Supplementary file 2 [file Table_1.pdf]

Promoter-luciferase SNP/indel variants

			rs1819333 trans-eQTL				Preferred variant			Alternate variant		Preferred vs Alt
SNP ID	Map info	Motif ID	Z score	p val	* DGE CD vs cnt	Gene Symbol	Preferred variant	seq match	p value	Alt variant	p value	pval_ratio
rs1044059	6:166956409	MA1596.1			-	ZNF460	C	GCCCTGGCGACCCGGG	1.60E-04	A	3.20E-03	20
rs16900967	6:166957202-166957220	MA1517.1	-2.08	0.037	+	KLF6	GCCCTCACCCAGCCCTGG	GCCCTCACCCA	4.10E-04	G	4.90E-03	11.95
		MA1653.1				ZNF148	GCCCTCACCCAGCCCTGG	TGGCCCTCACCC	4.20E-04	G	5.00E-03	11.9
rs1819333	6:166960059	MA0162.4				EGR1	G	CCGCGGCCCCGCCC	2.90E-06	C	4.20E-05	14.48
rs2149083	6:166957483	MA1511.1	-2.59	0.010		KLF10	C	CCGCCGCCCCG	8.30E-04	G	1.50E-02	18.07
		MA1515.1				KLF2	C	CGCCCCGCCCA	2.10E-07	G	4.40E-06	20.95
		MA1517.1	-2.08	0.037	+	KLF6	C	CCCGCCGCCCC	5.80E-05	G	6.60E-03	113.79
		MA1107.2				KLF9	C	CCGCCGCCCCGCCCAC	3.50E-04	G	4.30E-03	12.29
		MA0147.3			-	MYC	G	GGCCGCGGGTGC	7.30E-04	C	1.20E-02	16.44
		MA0746.2			+	SP3	C	CCCGCCGCCCCGC	1.00E-04	G	6.10E-03	61
		MA0527.1				ZBTB33	G	CACCCGCGGCCCCGC	8.70E-05	C	3.60E-03	41.38
		MA0694.1				ZBTB7B	C	GCACCCGCCGCC	7.20E-04	G	1.40E-02	19.44
		MA1653.1				ZNF148	C	CCCGCCGCCCCG	6.70E-06	G	1.60E-04	23.88
		MA1596.1			-	ZNF460	C	GCACCCGCCGCCCCGC	2.20E-05	G	3.20E-04	14.55
		MA0504.1	-2.08	0.037		NR2C2	C	CGGGTGCAGAGCCCG	6.00E-04	G	8.90E-03	14.83
rs2149084	6:166957500	MA1515.1				KLF2	G	GACAACGCCGG	5.60E-04	A	1.20E-02	21.43
rs2149092	6:166959490	MA1517.1	-2.08	0.037	+	KLF6	G	GACAACGCCGG	9.60E-04	A	9.80E-03	10.21
		MA1520.1			-	MAF	G	CTGCAGAGACAACGC	9.30E-05	A	3.90E-03	41.94
		MA1554.1	-3.11	0.002	-	RFX7	G	CGTTGTCTC	7.40E-04	A	4.10E-02	55.41
		MA1116.1			+	RBPJ	A	AGTGGGAAAA	2.90E-04	T	7.60E-03	26.21
		MA0473.3			+	ELF1	C	CAGGA**G**GAAGTGAC	2.20E-05	T	1.20E-03	54.55
		MA1483.1			+	ELF2	T	GTACAGGAAGAA	2.60E-04	C	9.80E-03	37.69
		MA0641.1	-3.43	0.001	+	ELF4	C	CAGGAGGAAGTG	1.70E-04	T	3.30E-03	19.41
		MA0028.2			+	ELK1	T	ACAGGAAGAA	6.10E-04	C	7.80E-03	12.79
		MA0759.1				ELK3	T	ACAGGAAGAA	4.70E-04	C	2.40E-02	51.06
		MA0076.2			-	ELK4	C	TCACTTCCTCC	8.10E-05	T	2.80E-03	34.57
		MA0098.3				ETS1	C	GGAGGAAGTG	3.50E-04	T	1.20E-02	34.29
		MA0645.1			+	ETV6	C	GGAGGAAGTG	3.30E-05	T	2.30E-03	69.7
		MA0475.2	-2.80	0.005	+	FLI1	T	ACAGGAAGAA	4.50E-04	C	6.00E-03	13.33
		MA0062.3				GABPA	T	ACTTCTTCCTGTAC	6.40E-05	C	1.20E-03	18.75
rs2149093	6:166959557	MA1508.1				IKZF1	T	AGTACAGGAAGA	6.30E-05	C	1.30E-03	20.63
		MA0508.3				PRDM1	C	CACTTCCTCCT	8.30E-04	T	1.30E-02	15.66
rs2757042	6:166957746	MA0080.5			+	SPI1	C	GTACAGGAGGAAGTGACAAG	1.60E-06	T	4.40E-05	27.5
rs2769346	6:166957511	MA0750.2	-3.43	0.001		ZBTB7A	C	AGGAGGAAGTGAC	1.00E-04	T	2.50E-03	25
		MA1522.1	-2.19	0.028		MAZ	C	GGCCCCTCGCT	1.10E-04	T	4.00E-03	36.36
		MA1653.1				ZNF148	C	GGCCCCTCGCTG	9.80E-05	T	4.40E-03	44.9
		MA1585.1			+	ZKSCAN1	G	GCAGTAGCTC	7.40E-04	T	7.40E-03	10
rs2149085	6:166957622	na				na	C/T	na	na	na	na	na

Supplemental Table 1. Regulatory Sequence Analysis Tool predicted motif disruption of transcription factor SNP/Indel variants in LD (R2>0.8) with index rs1819333 and regulatory rs2149092 variants. Trans-eQTL p values of TF expression vs RNASET2 risk vs non-risk carriage..
* differential TF gene expression in whole blood from CD vs non-IBD subjects (ref. 38)
